# Metrological Characterization and Comparison of D415, D455, L515 RealSense Devices in the Close Range

**DOI:** 10.3390/s21227770

**Published:** 2021-11-22

**Authors:** Michaela Servi, Elisa Mussi, Andrea Profili, Rocco Furferi, Yary Volpe, Lapo Governi, Francesco Buonamici

**Affiliations:** Department of Industrial Engineering, University of Florence, Via Santa Marta 3, 50139 Florence, Italy; michaela.servi@unifi.it (M.S.); elisa.mussi@unifi.it (E.M.); andrea.profili@unifi.it (A.P.); rocco.furferi@unifi.it (R.F.); yary.volpe@unifi.it (Y.V.); lapo.governi@unifi.it (L.G.)

**Keywords:** RealSense D415, RealSense D455, depth camera, RealSense L515, ISO 10360-13, device characterization, active stereo, LiDAR, performance comparison, reverse engineering

## Abstract

RGB-D cameras are employed in several research fields and application scenarios. Choosing the most appropriate sensor has been made more difficult by the increasing offer of available products. Due to the novelty of RGB-D technologies, there was a lack of tools to measure and compare performances of this type of sensor from a metrological perspective. The recent ISO 10360-13:2021 represents the most advanced international standard regulating metrological characterization of coordinate measuring systems. Part 13, specifically, considers 3D optical sensors. This paper applies the methodology of ISO 10360-13 for the characterization and comparison of three RGB-D cameras produced by Intel^®^ RealSense™ (D415, D455, L515) in the close range (100–1500 mm). ISO 10360-13 procedures, which focus on metrological performances, are integrated with additional tests to evaluate systematic errors (acquisition of flat objects, 3D reconstruction of objects). The present paper proposes an off-the-shelf comparison which considers the performance of the sensors throughout their acquisition volume. Results have exposed the strengths and weaknesses of each device. The D415 device showed better reconstruction quality on tests strictly related to the short range. The L515 device performed better on systematic depth errors; finally, the D455 device achieved better results on tests related to the standard.

## 1. Introduction

Once used only in applications that required high frame rates, depth cameras can nowadays be considered as a budget-option 3D optical coordinate measurement system. Big acquisition volumes, compact dimensions, low costs (w.r.t other professional 3D scanning systems), portability and easiness of use—i.e., the main features of such systems—have made RGB-D sensors the hardware of choice in several research and application fields.

From a technical perspective, RGB-D devices are optical sensors specifically designed to acquire 3D information from an observed scene; as their name suggests, they are capable of integrating the information provided by a 2D RGB image with additional depth information per pixel, creating a depth map of the scene. This study specifically refers to low-budget compact sensors, typically identified with the alternative name of “depth cameras”.

Pioneer products of this class of devices, such as the Microsoft Kinect [1], first commercialized in 2010, were essentially used as motion sensing and 3D tracking devices thus spreading in the fields of gaming and entertainment applications. As previously mentioned, continuous hardware and software development has enormously increased the area of applicability of these sensors. While acquisition speed, portability and compactness are distinctive features for the entire class of devices, different hardware is used as sensor, resulting in different operating principles. In particular, two main technologies can be identified: (i) Stereoscopic systems; (ii) Time-of-Flight (ToF) systems.

Stereoscopic systems base their interpretation of the scene on the comparison of two images acquired from two known and slightly different positions. Active lighting of the scene can—and usually is—used to add recognizable features to strengthen the depth computation and increase the robustness of the device. Projectors operating at different wavelengths can be introduced in the scene to add speckle patterns easily readable by the device. On the other hand, ToF systems rely on the measurement of the time required by a light signal emitted from the device to bounce off the objects and return to an optical sensor observing the scene. The type of operating principle evidently influences metrological performances as well as how the system tolerates different environment conditions.

As previously mentioned, several applications for these types of sensors can be identified [2,3,4,5,6,7,8,9]. 3D vision through depth cameras has been extensively applied in several fields such as: (1) robotics, to provide mechanical systems with a vision apparatus that need to interact with the environment [10,11] and for the acquisition and interpretation of indoor [12] and outdoor [13] environments; (2) autonomous driving applications [14] and (3) in the biomedical field with the ultimate goal of digitizing the anatomy of a patient [15,16], human modelling, recognition and tracking [17,18]. Due to their functioning principle, these sensors are widely applied in a series of scenarios where there is the need to mimic human vision, both in terms of Field-of-Vision (FoV) and ambient conditions of the work environment.

Until recently, applications that make use of depth cameras were interested mainly in their resolution, frame rate and range performances. The development of more accurate sensors has changed the scenario and has increased the importance that precision and accuracy have in the choice of a sensor: knowledge on the metrological performances of RGB-D devices became a significant issue. This is particularly true when working on close range. It is important to note that accuracy is typically proportional to the distance between sensor and object; accordingly, whenever users want to exploit sensors to their maximum, they usually need to work in close range.

Some studies in the scientific literature have addressed metrological characterization of depth cameras [19,20,21]. In [22], a comparison between three depth cameras has been presented; the authors perform a single test where each camera is required to acquire a flat wall at different distances; the authors evaluate the performances in terms of accuracy and precisions of the systems. Authors in [23] present a technological overview of ToF depth sensors that includes some RGB-D sensors. Specific test campaigns, focused to the analysis of the performance of a single sensor, have been proposed for several commercial sensors. The Intel^®^ RealSense™ Depth Camera D415 is reviewed in [24] with specific reference to close range (up to 1 m); the Intel^®^ RealSense™ L515 has been tested according to the VDI/VDE Guideline 2634 [25] in [26].

Achieving a replicable and meaningful description of the metrological performances of depth cameras is a goal whose importance is recognized by both the scientific and industrial communities. This is supported by the recent study, development and introduction of standards that regulate significant tests to be performed for a metrological evaluation of such sensors. VDI/VDE Guidelines have moved a first step in this direction by proposing the VDI/VDE 2634 guideline [25]. More recently, the ISO 10360-13:2021 has been introduced, and now represents the most advanced international standard for this type of evaluation.

Accordingly, this manuscript proposes the description of the metrological performances of three commercial RGB-D sensors. All three sensors are produced by Intel^®^ RealSense™ and represent the state-of-the-art in terms of technological development of this class of sensors. Realsense™ is a product line of depth-sensing devices commercialized by Intel^®^ (Intel, Santa Clara, CA, USA), specifically designed to give systems depth perceptions capabilities. Intel^®^ integrates both software and hardware products to assure the best acquisition and interpretation of depth data. All Realsense™ cameras come with an SDK that offers tools to support the acquisition process [27]. These tools are widely adopted and, to the best of the authors’ knowledge, are considered representative examples of the state-of-the-art of budget depth sensors. 

Traditionally, Realsense™ systems were typically built using the stereoscopy principle; recently, however, a new line of cameras, based on the ToF principle, were developed and commercialized. This article proposes a structured comparison between three devices that belong to the two families of sensors. Such analysis can be taken by the reader as an off-the-shelf tool to establish which sensor represents the best choice for their project. In order to guarantee the significatively and replicability of the comparison, the analysis has been carried out following the tests and procedures documented in the ISO 10360-13:2021 “Geometrical product specifications (GPS)—Acceptance and reverification tests for coordinate measuring systems (CMS)” and specifically refers to the Part 13 “Optical 3D CMS” of the standard. The analysis is carried out with specific reference to close range distances, with camera FoVs limited to a maximum depth of 1.5 m. 

Due to the high relevance, in the scenario of metrological characterization of optical devices, of the introduction of an international standard, a brief description of the new ISO standard is proposed in Section 2, in particular the authors report some key aspects on which the performed close range characterization was based. Section 3 describes the proposed characterization strategy; results are shown in Section 4 and finally discussed in Section 5.

## 2. The ISO 10360-13:2021 Standard

The ISO 10360-13 standard, published in 2021, specifies acceptance and reverification tests to verify the manufacturer’s claimed performances of an optical CMS. This standard is a real milestone in the context of the metrological characterization of 3D optical systems, which until now have not been addressed by any international standardization. Principles of optical measurement referenced in the standard include, for example, pattern projection, fringe projection and line-scanning systems, or similar, that provide single views without the assistance of external information regarding the position and orientation of the objects being scanned relative to the CMS.

The ISO 10360-13:2021 standard specifies the execution of four different tests in order to evaluate the performance of an optical device: Probing error (divided into Probing Size and Probing Form dispersion), Distortion error, Flat form distortion, Volumetric length measurement error in concatenated measurement volume. In addition to the tests, the document provides information about artifact sizes, artifact materials and environmental conditions to correctly perform the tests. 

The following is a summary description of the mandatory tests, specifying artifacts, scanning positions, and error computation. The principle of each test is to determine the maximum error committed by the optical sensor in the predefined measurement volume. Such measurement volume is considered to be divided into 8 voxels, and L_0_, referenced in the following, represents the maximum enclosed length—i.e., the maximum distance between two points included in the measurement volume.

### 2.1. Probing Characteristics

A calibrated spherical artifact that meets the following dimensions is expected for the probing test:

0.02*L_0_ < ф < 0.2*L_0_,

where ф is the diameter of the sphere.

**Figure 1:**
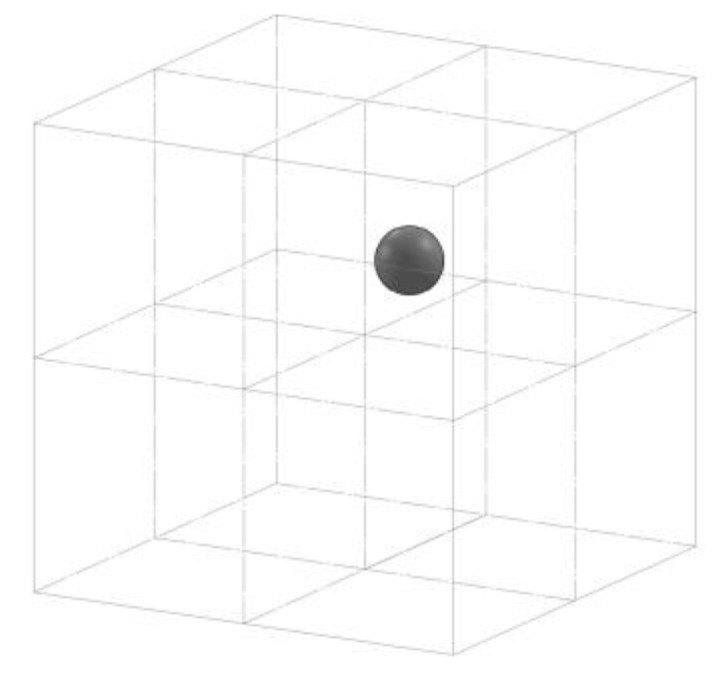
Example of spherical artifact acquired in a voxel of the measurement volume.

The artifact must be positioned and acquired in each of the 8 voxels of the measurement volume (Figure 1), each acquisition must be repeated 3 times and on each of them 2 errors are calculated: Probing form dispersion error (P_F_) and Probing size error (P_S_). P_F_ is defined as the minimum width of the spherical shell enclosing the measured points; the shell is built considering two concentric spheres embracing, in the volume incapsulated by the two spheres, all the acquired points. P_S_ is the difference between the calculated diameter associated with the unweighted Gaussian sphere and the true diameter. P_F_ and P_S_ are defined as the maximum errors obtained in the 24 performed acquisitions.

The corresponding multiview errors are computed from the registration and fusion of all measured points into a single point cloud representing the full artifact.

### 2.2. Distortion Characteristics

An artifact consisting of two spheres calibrated for the diameter and the center-to-center length is required for the distortion test:

L_P_ > 0.3*L_0_; 0.02*L_0_ < ф < 0.2*L_0_, where ф is the diameter of the spheres and L_P_ is the centre-to-centre distance. 

**Figure 2:**
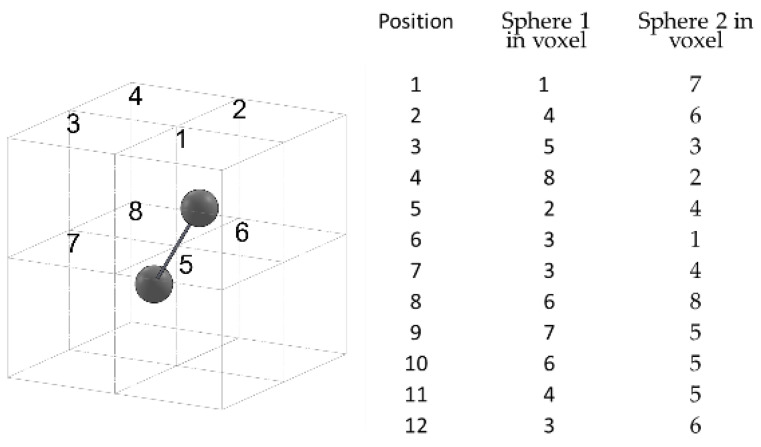
Ball bar positions provided by the standard for the distortion error measurement.

To measure the distortion error, the artifact is acquired at 12 positions within the measurement volume, shown in Figure 2. Each acquisition is repeated 3 times and the distortion error D, defined as the difference between the measured and true center-to-center distance, is calculated on each. D is defined as the maximum error obtained in the 36 acquisitions.

The corresponding multiview error is computed from the registration and fusion of all measured points into a single point cloud representing the full artifact.

### 2.3. Flat Form Distortion Error

A calibrated planar artifact with the following dimensions is considered for the flat form distortion test:

L_MAX_ ≥ 0.5*L_0_ and L_MIN_ ≥ 0.1*L_0_, where L_MAX_ and L_MIN_ are the longest and shortest lengths of the plane, respectively. 

The standard also states that, in the event that a plane with the required L_MIN_ is not available, an artifact with a shorter L_MIN_ may be used as long as a value greater than or equal to 50 mm is ensured. 

**Figure 3:**
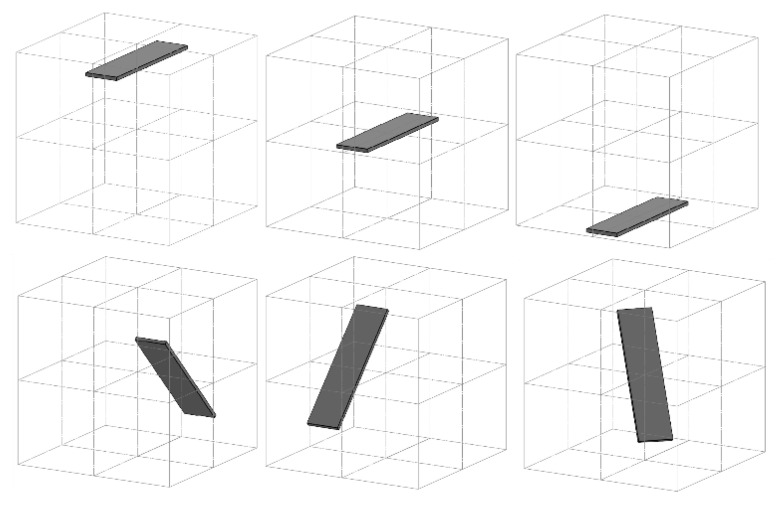
Planar artifact positions provided by the standard to measure the flat distortion error.

To measure the flat form distortion error the artifact is acquired at 6 different positions, shown in Figure 3. On each acquisition, the flat form distortion error F is calculated, defined as the shortest distance between two parallel planes encompassing the scanned data.

The corresponding multiview error is computed from the registration and fusion of all measured points into a single point cloud representing the full artifact.

### 2.4. Volumetric Length Measurement Error in Concatenated Measurement Volume

This test is performed in the concatenated measurement volume, defined as the measurement volume obtained by moving the sensor and recording acquisitions. The longest length inside the concatenated measurement volume must be at least twice as long as the longest length inside the sensor measurement volume (L_0_).

**Figure 4:**
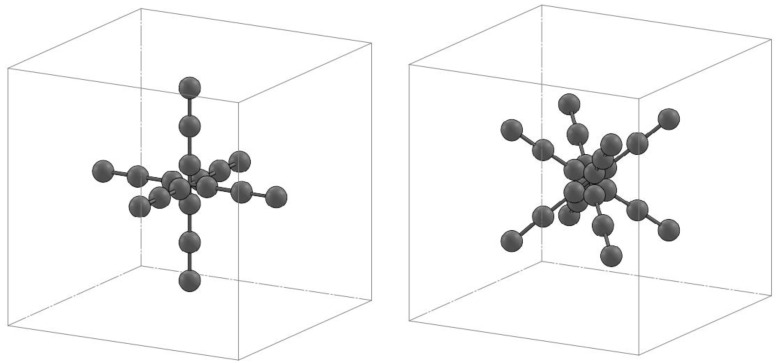
Example of the artifact composed of 5 center-to-center length acquired in the concatenated measurement volume.

An artifact consisting of at least 5 center-to-center lengths is defined for this test, where the longest length must be at least 66% of the diagonal of the concatenated measurement volume. The artifact must be placed in 7 positions in the measurement volume of which 4 must be on the diagonals, an example is shown in Figure 4; each length must be measured 3 times, for a total of 105 measurements. 

This test was not included in the characterization, partly because it requires the realization of complex artifacts (not easily accessible on the market given the high dimensions of the required spheres) and the availability of large environments for acquisition, and because the authors believe that the other tests can provide a sufficiently accurate characterization of the devices.

## 3. Materials and Methods

**Figure 5:**
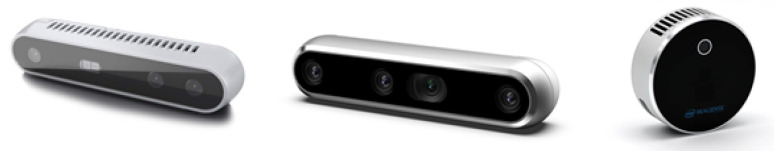
Three depth cameras under review in this study. From left to right: Intel^®^ RealSense™ Depth Camera D415, Intel^®^ RealSense™ Depth Camera D455, Intel^®^ RealSense™ LiDAR Camera L515.

In this study three 3D optical sensors with comparable characteristics and similar application areas were analyzed. The three optical sensors, depicted in Figure 5, are produced by Intel^®^ RealSense™. Commercial names of the three devices are D415, D455 and L515.

Both D455 and D415 share a number of key features, as they are both part of the D400 series. Both systems are Active Stereoscopic, have identical framerates and similar physical structure. 3D knowledge is, in both cases, obtained performing a comparison of two RGB images acquired by two sensors and evaluating the disparity map. Both sensors introduce active lighting in the scene and integrate RGB data with a projected IR pattern that is invisible to the human eye. Main differences between the two systems lie in the type of shutter used (rolling for the D415, global for the D455) and in the FoVs. D415 is characterized by a smaller FoV and a maximum depth in the ideal range of 2 m; D455, on the other hand, has a maximum ideal acquisition distance of 6 m.

The L515, on the other hand, is a LiDAR camera; it projects an infrared laser at 860 nm wavelength as active light source. 3D data is obtained evaluating the time required to the projected signal to bounce off the objects of the scene and come back to the camera. Its efficacy is influenced by the quality and characteristics of the materials of the measured objects. It has a maximum ideal acquisition distance of 9 m and a much slower depth fps rate when compared with D415 and D455 (30 fps vs. 90 fps). 

Detailed information on the main features of the three devices are reported in Table 1.

**Figure 6:**
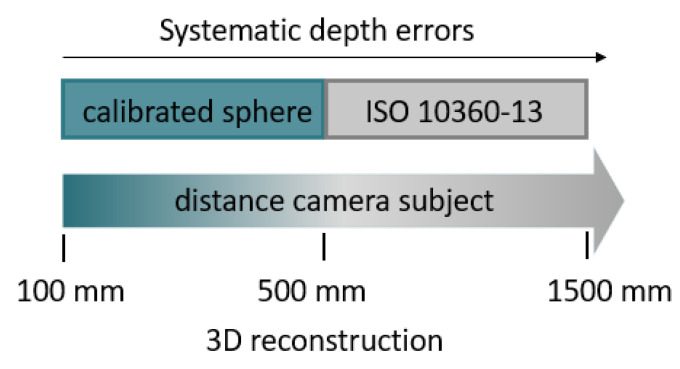
Adopted metrological characterization framework. The Figure shows which tests were performed depending on the acquisition distance.

Figure 6 shows the applied characterization framework and summarizes the tests performed depending on the acquisition distance.

**Figure 7:**
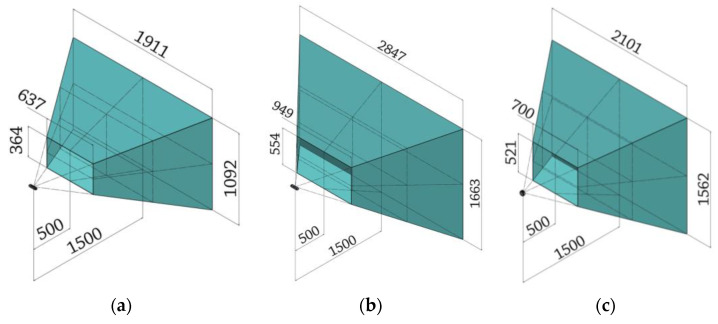
Field of view of the three devices in the range 500–1500 mm. From left to right: (**a**) D415, (**b**) D455, (**c**) L515.

In the 500–1500 range the devices were tested following the guidelines of the ISO standard. This range produces the measurement volumes shown in Figure 7 for each device under consideration.

Differently from what is shown in the standard, in which the measurement volume is assimilable to a parallelepiped, in the case of the Intel RealSense devices under examination the measurement volume is a frustum. This determines a not negligible difference between the area framed by the device at the closest distance to the camera and the most distant one; subsequently, it complicates the shape of the artifacts to be acquired, as they need to be fully enclosed even in the four proximal voxels. Despite the significantly different shape of the measurement volume, preliminary tests have shown that in the acquisition range 500–1500 mm it is possible to dimension the artifacts according to the standard and ensure their full acquisition in the required positions. 

The performances of the cameras in the remaining part of the close range (100–500 mm) were evaluated by acquiring a specific artifact consisting of a calibrated sphere. Finally, systematic depth errors, namely the depth offset and systematic non-planarity, were evaluated over the entire range, comparing the performance of the three devices under investigation. 

The RealSense devices are controlled through the proprietary SDK. The software interface supports several depth presets that can be selected based on the end use. Among the available presets, the Default configuration provides the most generic camera parameters by not applying particular filters to the acquired data. Therefore, with the aim of providing a complete characterization of the camera and allowing a comparison with other similar devices, while keeping the analysis as general as possible, in this work, the Default configuration was considered as a starting point from which to vary only a subset of critical parameters. 

The tests were performed by setting the value of depth unit to 10^−3^ for the D415 and D455 cameras in order to obtain the best depth quantization possible, the value of depth unit for the Lidar L515 is not modifiable and is set to 0.00025. In order to ensure maximum performance of the devices, in all cases it was chosen to set the resolution to the maximum possible value (for D415 equal to 1280 × 720, for D455 equal to 1280 × 720 and for L515 equal to 1024 × 768). 

As a result, the comparison of the devices has evaluated and compared the cameras at their maximum performance.

### 3.1. Experimental Setup

#### 3.1.1. Calibrated Sphere (100–500 mm)

**Figure 8:**
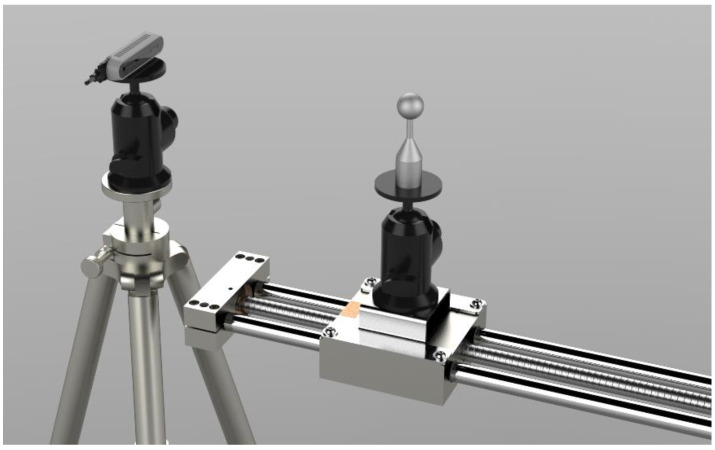
Acquisition setup for the very-close range test as in [24].

For the acquisition of objects in the very-close range (100 mm up to 500 mm), characterization is performed using a calibrated sphere of diameter 25.4 mm that is placed at progressive distances from the camera with a fixed step of 100 mm. The artifact is fixed on a mechanical linear guide with a 4 mm pitch maneuvering screw, as exemplified in Figure 8, which ensures positioning accuracy and repeatability. The artifact is moved and acquired at 4 positions (150 mm, 250 mm, 350 mm, and 450 mm).

The points acquired on the surface of the sphere are isolated for each acquisition; the diameter of the best-fit sphere is then computed and compared with the ground truth to extract the parameter of interest for the characterization (i.e., differences between the two values). 

In order to acquire depth in this range, certain parameters must be modified: with regard to devices with active stereo technology, the disparity shift values must be modified. Active stereo systems evaluate depth as the proportional inverse of the pixel disparity of the right IR image to the left IR image, where the pixel disparity is evaluated along the rectified epipolar lines [28]. This depth, called disparity shift, can in fact be varied to change the acquisition range. As default configuration this value is set to 0 to cover an acquisition range from minZ to infinity, where minZ is given by the distance of the left and right imagers (namely the baseline); by varying this value it is possible to decrease minZ while creating an upper limit to the acquired depth, maxZ. In order to perform the test in the very close range the disparity shift values were set to allow the acquisition at four fixed distances from the camera. Specifically, these values were chosen following the procedure detailed in [24]. For the L515 sensor it is necessary to modify the minimum distance parameter in order to perform an acquisition closer to the device. 

#### 3.1.2. ISO 10360-13:2021 Standard (500–1500 mm)

The three devices are characterized in the measuring volume 500–1500 mm by following the tests predefined in the standard described in Section 2. 

In order to dimension the artifacts according to the standard, the diagonals of the measurement volumes were calculated, which permitted the identification of an acceptable range of the dimensions of each artifact. Possible sizes of the derived artifacts for characterization of each device in accordance with ISO 10360-13 are shown in Table 2.

**Figure 9:**
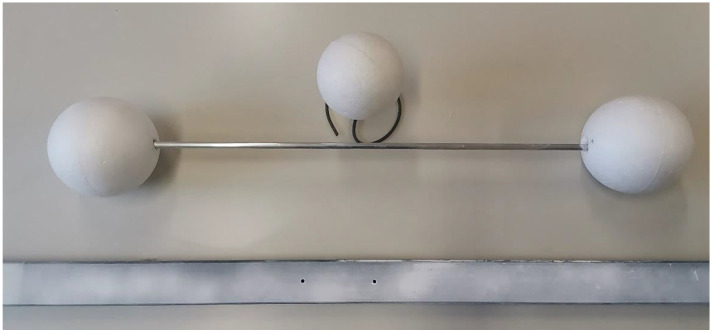
Artifacts selected for device characterization according to the standard ISO 10360-13:2021.

Once the feasible ranges were defined, artifact sizes were established as follows: starting from the values suggested by the standard, the artifacts chosen for carrying out the test had the following dimensions: the single sphere had a diameter (ф) of 142.28 mm, the distance between the centers of the two spheres of the ball-bar (L_P)_ was 752.15 mm, and the plane for the flatness error was 1250 mm long and 50 mm high. The artifacts used in this study are depicted in Figure 9. The selected artefacts were all characterized by a white, opaque and smooth surface, obtained with the application of a white developer spray. 

These values were measured with a high-precision professional scanner, the Romer AbsoluteArm 7520 SI/SE equipped with a RS1 optical scanner (Hexagon Metrology S.p.A., Turin, Italy), which has an accuracy of ±0.063 mm. Its measurements can, therefore, be considered as ground truth for the purpose of this work.

#### 3.1.3. Systematic Depth Errors (100–1500 mm)

**Figure 10:**
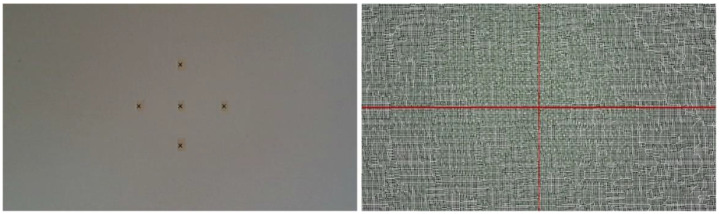
Acquisition setup to ensure camera-plane perpendicularity during the systematic depth error evaluation test.

One of the most common systematic errors for RGB-D devices is the so-called “inhomogeneous distance”, i.e., the set of possible errors that can result from acquisitions at different distances from the sensor. To study these errors, it is necessary to place the sensor perpendicular to a planar surface and acquire it at different distances. Specifically, the distances considered in this study are in the range 100–1500 mm, with a 100 mm step. For this purpose, the first step is to place a linear guide perpendicular to the reference plane. The guide is positioned with its axis perpendicular to the reference plane. To ensure correct positioning, orthogonal cross references were inserted as markers on the planar surface; these references are used to verify their alignment with the cross drawn in the center of the image as the sensor moves away from the surface (see Figure 10).

The objective of this test was to evaluate two types of errors: systematic non-planarity errors and offset errors on depth. The first one was evaluated by referring to a ground truth plane (with a certified flatness of 50 μm), to verify the flatness of the obtained scan. The second was analyzed by studying the deviation between the obtained scan and the ground truth plane.

#### 3.1.4. 3D Object Reconstruction (~500 mm)

The last test concerns the three-dimensional reconstruction of objects. In line with two previous works on characterization of Intel RealSense sensors [24,29], it was decided to use two different objects: a smooth free-form object (~200 mm high and ~70 mm wide) and a 3D tangram object (bounding box with dimensions of 150 mm high and 150 mm wide). To perform the acquisition, the objects were rotated with respect to the stably positioned sensor.

To provide an effective and easily replicable evaluation, the sensors were used in the default configuration, with the only modified parameter being the depth unit (for D415 and D455), set to the best achievable resolution. To ensure the best performance, the sensor-object distance was set to the smallest feasible value of approximately 500 mm. For both objects, ground truth data was acquired with the Romer Absolute Arm 7520 SI/SE and RS1 optical scanner and compared with the 360° reconstruction obtained by acquiring the objects with the three sensors under consideration.

## 4. Results

All of the results listed in this section were obtained by acquiring 3D data for each test using the Depth Quality Tool software provided by Intel RealSense, subsequently processed using Geomagic Design X^®^ 3D modeling software [30]. In detail, the points acquired on the artifact surfaces are isolated for each acquisition, removing 8% of edge points as described in [29]; subsequently best-fit operations of planar and spherical primitives are computed (using the tools made available by the software) as well as deviation analysis operation, to compare the extracted information with the nominal values of the artifacts to obtain the parameter of interest for the characterization. With regard to the 3D reconstruction test, in order to create the three-dimensional model of each acquired object, after removing 8% of the edges from each acquisition, it was necessary to perform an alignment process consisting of two phases, a first coarse alignment based on the manual selection of corresponding points and a fine alignment phase using the Iterative Closest Point (ICP) algorithm.

### 4.1. Calibrated Sphere (100–500 mm)

Table 3 shows the results of the tests performed in the very close range (100–500 mm), i.e., the difference between the true diameter of the calibrated sphere (equal to 25.4 mm) and the one measured by the three sensors, averaged across the four positions. 

**Figure 11:**
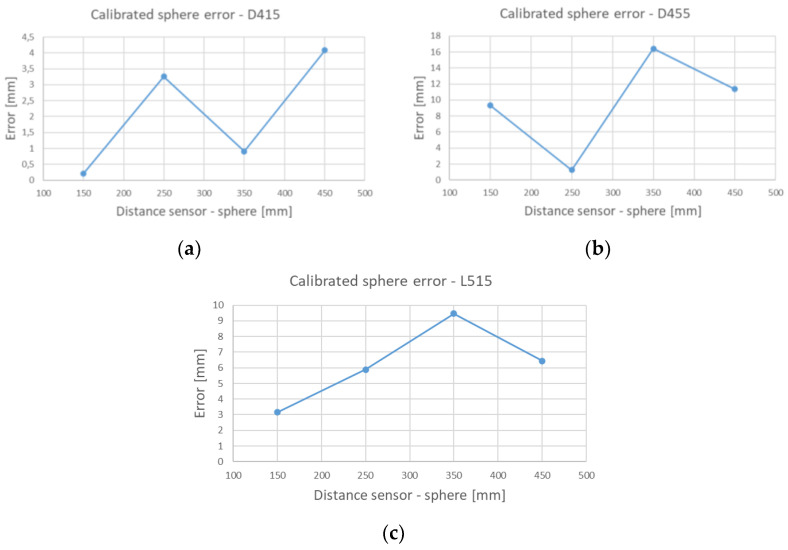
Error of calibrated sphere diameter estimation in the range 100–500 mm for (**a**) the D415, (**b**) the D455 and (**c**) L515 devices.

To appreciate the error trend as the distance between the sensor and the acquired sphere increases, Figure 11 reports this parameter for the three examined devices.

### 4.2. Close Range (500–1500 mm)

#### 4.2.1. Probing Errors

**Figure 12:**
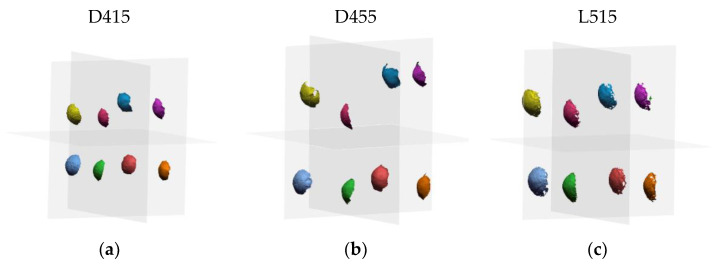
Acquisitions of the sphere artifact performed by the three sensors according to the ISO 10360-13 standard. (**a**) D415 data; (**b**) D455 data; (**c**) L515 data.

Figure 12 shows the acquisitions of the sphere artifact performed by the three sensors in the 8 positions indicated by the standard within the measurement volume.

Table 4 reports the average values of the probing size error calculated across all positions for each sensor as the difference between the true value of the sphere diameter and the measured value.

Table 5 shows the average values of the probing form calculated for each sensor, that is, the average of the differences between the maximum and minimum distances of the points of the measured surface of the i-th sphere with respect to the i-th surface of the best-fit sphere.

#### 4.2.2. Distortion Characteristics

**Figure 13:**
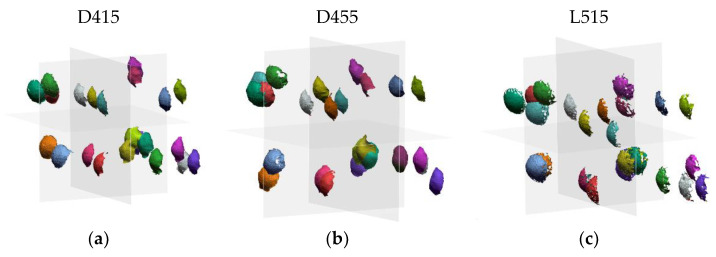
Ball bar acquisitions performed by the three sensors according to the ISO 10360-13 standard. (**a**) D415 data; (**b**) D455 data; (**c**) L515 data.

Figure 13 shows the ball bar acquisitions performed by the three sensors in the 12 positions indicated by the standard within the measurement volume.

Table 6 shows the average values of the distortion error calculated for each sensor, i.e., the average difference between the true and the measured centre-to-centre distance of the ball bar.

#### 4.2.3. Flat Form Distortion Error

**Figure 14:**
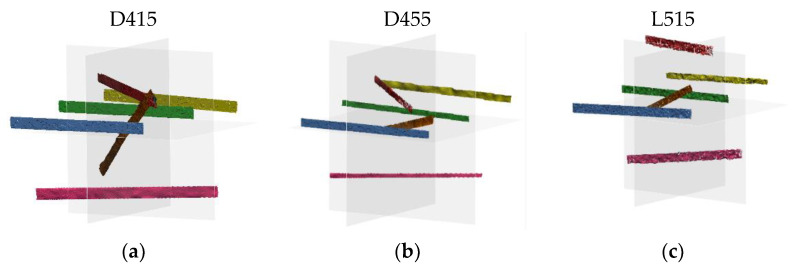
Plane acquisitions performed by the three sensors according to the ISO 10360-13 standard. (**a**) D415 data; (**b**) D455 data; (**c**) L515 data.

Figure 14 shows the plane acquisitions performed by the three sensors in the 6 positions indicated by the standard within the measurement volume.

Table 7 shows the average values of the flat form distortion error calculated for each sensor, that is, the average of the differences between the maximum and minimum distances of the points of the measured surface of the i-th plane with respect to the i-th surface of the best-fit plane.

### 4.3. Systematic Depth Errors (500–1500 mm)

**Figure 15:**
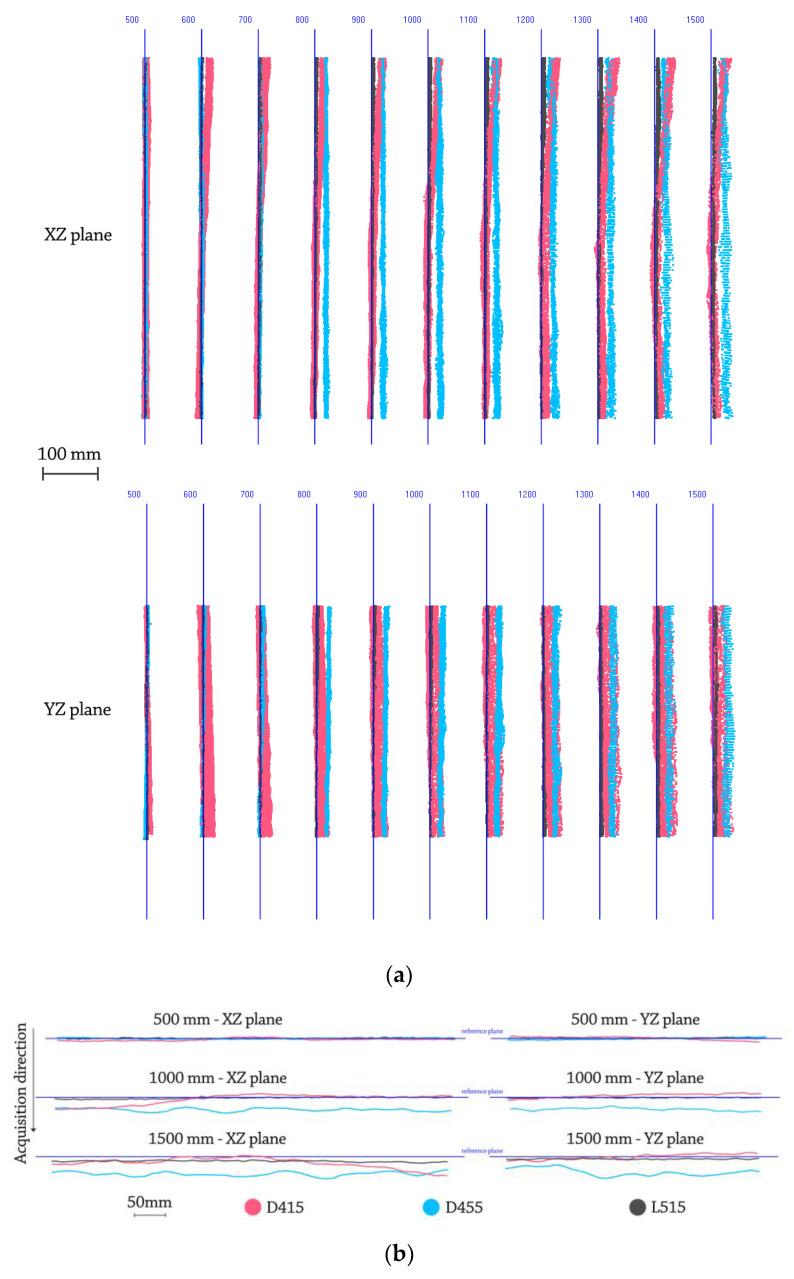
(**a**) superior view (top) and lateral view (bottom) of the acquisition performed for the evaluation of the systematic depth errors; (**b**) detail of a few acquired planes simplified through lines that interpolate points in correspondence of two orthogonal planes (XZ plane, YZ plane) that intersect the optical axis of the camera.

The systematic depth errors evaluated in this section are: (i) offset errors on the depth and (ii) systematic non-planarity errors. The first one was qualitatively analysed considering the differences in the Z-distances between scanned planes and the ground truth planes (with Z being the optical axis of the camera). As previously mentioned, data is acquired with a fixed step equal to 100 mm. Figure 14 shows the result of such test (in light red D415, in light blue D455 and in grey L515). Specifically, in Figure 15a it is possible to observe the offset error from the lateral view, while Figure 15b shows the acquired planes from the superior view, helpful to examine in addition to the depth offset the planarity deformation.

The systematic non-planarity error was evaluated by referring to a ground truth plane (with a certified flatness of 50 μm), to verify the flatness of the obtained point cloud. 

Table 8 shows the errors, averaged across all acquisitions, in terms of (1) mean distance and standard deviation from the best-fit plane and (2) range, defined as the difference between the maximum and minimum distance of the acquired plane from the best-fit plane. 

**Figure 16:**
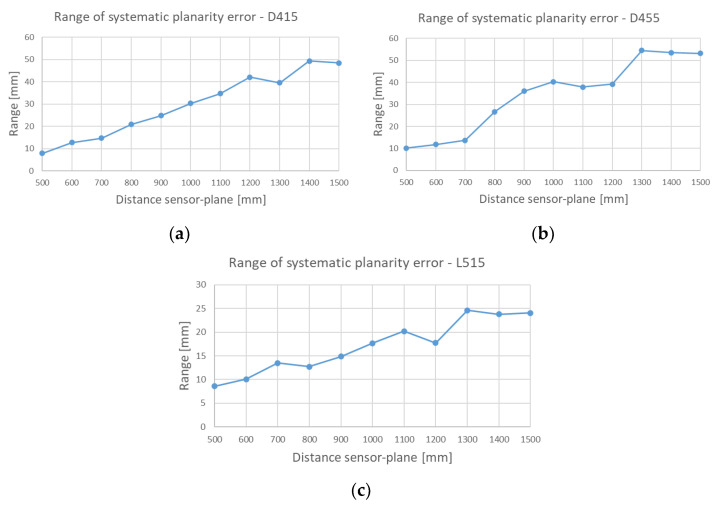
Error for the planarity estimation test in the range 100–1500 mm for (**a**) the D415, (**b**) the D455 and (**c**) L515 devices.

To appreciate the error trend as the distance between the sensor and the acquired plane increases, Figure 16 reports this parameter for the three examined devices.

### 4.4. 3D Object Reconstruction (~500 mm)

**Figure 17:**
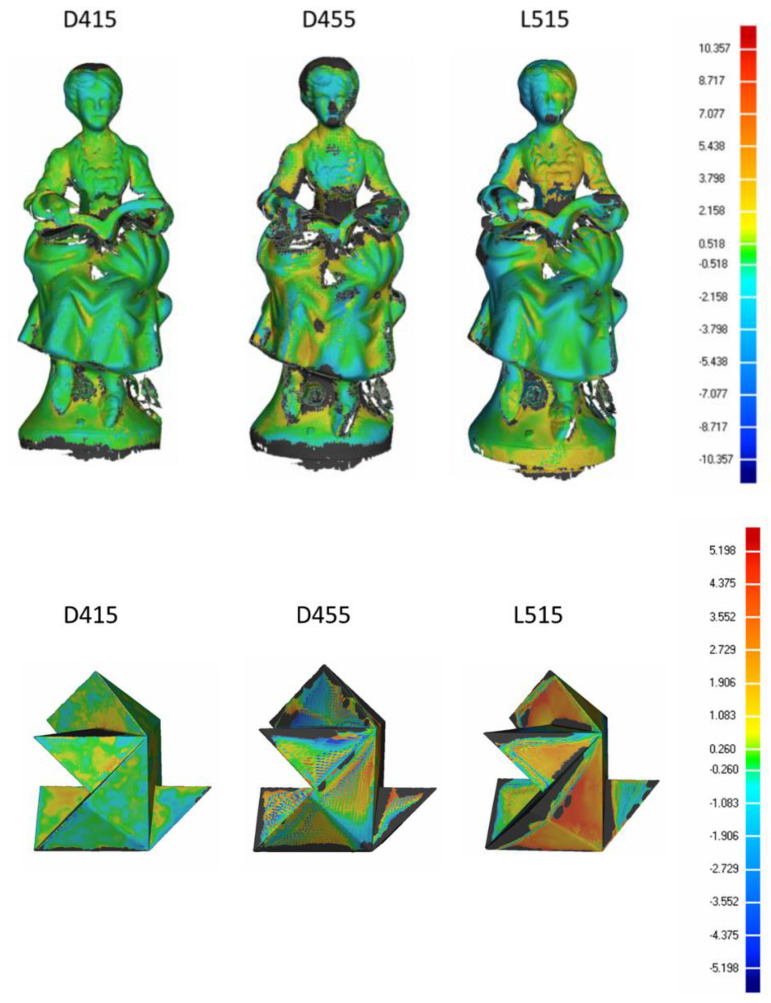
Deviation results of the two 3D reconstructions (free form statue object and tangram object). From left to right, the figure shows the data obtained with D415, D455 and L515 sensors. The colour scale describes the distance between the reference model and the acquired model in mm; positive values are marked with colours from green to red, negative values are marked with colours from blue to green.

Figure 17 shows the Euclidean distances between the acquired artifacts and the ground truth, where the gray areas are not considered in the deviation analysis (in these areas the data may be missing or too distant from the ground truth).

Table 9 and Table 10 show the reconstruction errors in terms of mean distance, standard deviation and range of deviation between the reconstructed data and ground truth.

## 5. Discussion and Conclusions

Based on the results reported above, the authors want to discuss and compare the performances of the devices under investigation to raise awareness of the scientific community on the use of these devices for metrological applications. This analysis has to be seen in the context of the use of low-cost commercial devices tested off the shelf, thus without making any changes to the factory characteristics (such as, for instance, recalibration). This means that different devices can obtain slightly different performances from those reported in this work, therefore the interpretation of results must take into account the interchangeability of devices, even belonging to the same model. The tests were carried out indoors, in a controlled brightness environment; all artifacts were properly made opaque to reduce effects that could penalize a specific depth technology over the others.

Analyzing the calibrated sphere test (performed in the very close range 100–500 mm), it emerges that the best average result is obtained by the D415 device (error equal to 2.1 mm), while the worst result is reported by the D455 device with a value equal to 9.58 mm. These results can be explained by the different destination of use of the devices, in fact the D455, while using the same technology of his older sister D415, is intended for a deeper vision and has a wider baseline (95 mm of D455 compared to 55 mm of D415).

With regard to the tests carried out in the 500–1500 mm range according to the standard, the lowest P_S_ error is reached by the D455 device with a mean value of 2.47 mm and std 1.42 mm. The lowest P_F_ error is obtained with the D415 device with a mean value of 7.93 mm and std 2.08 mm; the best D is obtained with the D455 device with a mean value of 6.83 mm and std 5.78 mm. Finally, for FD the best result is obtained with the D455 with a mean value of 16.05 mm and std 8.33 mm. The D455 allows the obtainment of lower average errors in most of the performed tests and also in this case the acquisition range can be considered an influential factor on the results. In fact, from comparing the obtained results with similar tests, even if performed with another standard in [24], it can be observed how an increase in the range of acquisition can negatively affect the performance of the D415 device. Finally, it is worth noting that the performance of the L515 device is (for all the performed tests) in an average range between the performance of the D415 and D455 devices, thus showing intermediate performance between the two.

Figure 15 shows in light red the planes acquired by D415, in light blue by D455 and in grey by the device L515; from a first qualitative analysis it can be observed that for all three devices there is a progressive shift of the acquired plane with respect to the real position. Moreover, the device L515 is less prone to the systematic depth displacement and to the “twist effect” maintaining a regularity of the acquired points with respect to the Z axis (appreciable in Figure 15b). As far as the flatness tests are concerned, the average values of the three devices are comparable, while from the point of view of the range that encloses 100% of the acquired data, the L515 device achieves the best result (range 17.06 mm) while the worst range (34.24 mm) is measured with the D455 device. The best performance of the L515 device can be linked to its ability to contain the twist effect that then limits high peaks of data.

Regarding the last test the average and range values obtained by the devices for both the statue and tangram object are comparable, although the reconstruction of the D415 device has more valid points. Regarding the 3D reconstruction performance of the D455 and L515 devices, no substantial differences are found.

In conclusion, this work has seen the metrological characterization of three RGB-D devices of the Intel RealSense family, namely D415, D455 and L515 with the aim of comparing their performances under different aspects. The study involved a wide range of qualitative and quantitative tests, some in line with tests previously performed by the authors in other works, others in line with the new standard for optical devices, the ISO 10360-13:2021. The tests performed are consistent across the three devices despite using different technologies to obtain the 3D coordinates of the observed scene.

Overall, the tests carried out have exposed the strengths and weaknesses of each device: in tests strictly related to the short range (calibrated sphere and 3D reconstruction) a better quality of reconstruction has emerged from the D415 device. With regard to the tests on systematic errors of depth a better ability in the representation of planar surfaces by the L515 device emerges; finally, the tests related to the standard underlined a higher ability of the D455 device.

## Figures and Tables

**Table 1:** Main features of the three devices under investigation: D415, D455 and L515. Data obtained from Intel^®^ RealSense™ website. Depth accuracy refers to errors computed in the evaluation of the Z coordinates of the acquired points with respect to the distance from the sensor.

	D415	D455	L515
Use Environment	Indoor/Outdoor	Indoor/Outdoor	Indoor
Depth Technology	Stereoscopic	Stereoscopic	LiDAR
Image Sensor Technology	Rolling Shutter	Global Shutter	Laser Scanning
Depth FOV (H × V)	65° × 40°	87° × 58°	70° × 55°
Depth Resolution	Up to 1280 × 720	Up to 1280 × 720	Up to 1024 × 768
Depth Accuracy	<2% at 2 m^2^	<2% at 4 m^2^	~5 mm to ~14 mm thru 9 m^2^
Depth Frame Rate	Up to 90 fps	Up to 90 fps	30 fps
RGB Sensor Technology	Rolling Shutter	Global Shutter	Rolling Shutter
RGB Sensor Resolution	2 MP	1 MP	2 MP
RGB Frame Rate and Resolution	1920 × 1080 at 30 fps	1280 × 800 at 30 fps	1920 × 1080 at 30 fps
RGB Sensor FOV (H × V)	69° × 42°	90° × 65°	70° × 43°
Inertial Measurement Unit	No	Yes	Yes
Minimum Depth Distance at Max Resolution	~45 cm	~52 cm	~25 cm
Ideal Range	0.5 m to 3 m	0.6 m to 6 m	0.25 m to 9 m
Dimensions (L × D × H)	99 × 20 × 23 mm	124 × 26 × 29 mm	61 × 26 mm

**Table 2:** Reference limits for sizing artifacts according to the standard’s guidelines.

	L_0_ mm	L_P_ mm	ф min mm	ф max mm	Plane min Length mm
**D415**	2414.8	724.4	48.3	483	1207.4
**D455**	1775.8	532.7	35.5	355.2	887.9
**L515**	2011.2	603.4	40.2	402.2	1005.6

**Table 3:** Average errors obtained in the very-close range test.

	D415	D455	L515
Average error	2.1 mm	9.58 mm	6.23 mm

**Table 4:** Average error and standard deviation of the P_S_ error.

	D415	D455	L515
Average P_S_	mean 5.62 mm std 3.11 mm	mean 2.47 mm std 1.42 mm	mean 3.48 mm std 1.37 mm

**Table 5:** Average error and standard deviation of the P_F_ error.

	D415	D455	L515
Average P_F_	mean 7.93 mm std 2.08 mm	mean 12.33 mm std 2.43 mm	mean 11.46 mm std 2.42 mm

**Table 6:** Average error and standard deviation of the D error.

	D415	D455	L515
Average D	mean 18.12 mm std 7.41 mm	mean 6.83 mm std 5.78 mm	mean 8.01 mm std 1.97 mm

**Table 7:** Average error and standard deviation of the FD error.

	D415	D455	L515
Average FD	mean 23.16 mm std 7.85 mm	mean 16.05 mm std 8.33 mm	mean 65.62 mm std 27.48 mm

**Table 8:** Average error, standard deviation and error range of the systematic depth error.

	D415	D455	L515
Systematic depth error	mean 0.62 mm std 4.57 mm range 29.57 mm	mean −0.7 mm std 4.25 mm range 34.24 mm	mean −0.4 mm std 1.88 mm range 17.06 mm

**Table 9:** Average error, standard deviation and error range of the 3D reconstruction error for the statue object.

	D415	D455	L515
3D reconstruction statue	mean −0.04 mm std 1.78 mm range 20.85 mm	mean 0.18 mm std 2.27 mm range 20.55 mm	mean −1.07 mm std 2.8 mm range 20.94 mm

**Table 10:** Average error, standard deviation and error range of the 3D reconstruction error for the tangram object.

	D415	D455	L515
3D reconstruction tangram	mean 0.11 mm std 0.9 mm range 10.37 mm	mean 0.16 mm std 2.29 mm range 10.39 mm	mean 0.48 mm std 2.61 mm range 10.5 mm
